# Ramelteon suppresses astrocytic senescence via MT1-cAMP axis to protect dopaminergic neurons

**DOI:** 10.3389/fnagi.2026.1974509

**Published:** 2026-10-26

**Authors:** Chao Gu, Yajing Chen, Xiaohuan Du, Fang Li, Shuwei Yuan, Wenjing Wang, Ping Guo, Shikai Wang, Mei Wang

**Affiliations:** 1 Department of Pharmacy, Children's Hospital of Soochow University, Suzhou, China; 2 Department of Mental Health, Children's Hospital of Soochow University, Suzhou, Jiangsu, China

**Keywords:** astrocytic senescence, cAMP, mitochondrial ROS, MT1 receptor, Parkinson’s disease, Ramelteon

## Abstract

**Introduction:**

Astrocytic senescence is increasingly recognized as a critical contributor to the pathogenesis of Parkinson’s disease (PD). Ramelteon, a clinically approved MT1/MT2 melatonin receptor agonist, has demonstrated neuroprotective effects in various neurological disorders. However, whether Ramelteon can inhibit astrocytic senescence and the underlying mechanisms remain unknown.

**Methods:**

An *in vivo* MPTP-induced mouse model of PD was used to evaluate the therapeutic potential of Ramelteon. An *in vitro* long-term culture-induced replicative senescence model (40 days *in vitro*, DIV) and MPP^+^-induced premature senescence model in primary astrocytes were employed. Immunoblotting, real-time quantitative PCR, immunofluorescence, SA-β-gal staining, and immunohistochemistry were performed to assess astrocytic senescence, dopaminergic neuronal survival, and motor function.

**Results:**

Ramelteon treatment significantly attenuated MPTP-induced dopaminergic neuronal loss in the substantia nigra compacta (SNc) and improved motor deficits in mice. *In vitro*, Ramelteon markedly suppressed both replicative and MPP^+^-induced astrocytic senescence, as evidenced by decreased p16^Ink4a^ and SA-β-gal activity, restored LaminB1 expression, and reduced senescence-associated secretory phenotype (SASP) factors (*IL-6*, *IL-1β*, *CXCL10*, and *MMP9*). Mechanistically, knockdown of *Mtnr1a* (encoding MT1), but not *Mtnr1b* (encoding MT2), largely abolished the anti-senescence effects of Ramelteon. MT1 activation inhibited adenylate cyclase (AC) activity and reduced intracellular cyclic adenosine monophosphate (cAMP) levels. Notably, senescent astrocytes exhibited elevated cAMP levels, and the cAMP inhibitor SQ22536 mimicked while the cAMP analog dbcAMP reversed the anti-senescence effects of Ramelteon. Furthermore, Ramelteon reduced mitochondrial ROS (mitoROS) production in senescent astrocytes through MT1-cAMP signaling.

**Discussion:**

Our findings demonstrate that Ramelteon inhibits astrocytic senescence through MT1 receptor-mediated cAMP suppression and mitochondrial ROS reduction, thereby protecting DA neurons in a PD mouse model. This study identifies Ramelteon as a potential therapeutic agent for PD by suppressing astrocytic senescence through MT1 activation.

## 1 Introduction

Parkinson’s disease (PD) is the second most prevalent neurodegenerative disorder, characterized by the progressive loss of dopaminergic (DA) neurons in the substantia nigra compacta (SNc) (Ben-Shlomo et al., 2024). Although the etiology of PD remains elusive, accumulating evidence indicates that aging is the predominant risk factor (Rodriguez et al., 2015; Collier et al., 2017). Cellular senescence, a hallmark of aging, has emerged as a critical contributor to age-related neurodegenerative diseases, including PD (Wang et al., 2024).

Astrocytes, the most abundant glial cells in the central nervous system (CNS), play essential roles in maintaining neuronal homeostasis, providing metabolic support, and regulating synaptic function (Patani et al., 2023). During aging or under pathological conditions, astrocytes undergo senescence, characterized by irreversible cell cycle arrest, upregulation of senescence markers such as p16^Ink4a^, downregulation of LaminB1, and acquisition of a senescence-associated secretory phenotype (SASP) (Cohen and Torres, 2019). Senescent astrocytes secrete excessive pro-inflammatory cytokines (*IL-6*, *IL-1β*, *CXCL10*, and *MMP9*), which are detrimental to neighboring neurons (Xia et al., 2020; Jiang et al., 2023; Chen et al., 2024). Importantly, senescent astrocytes have been identified in the SNc of PD patients and animal models (Simmnacher et al., 2020; Jiang et al., 2023), suggesting that astrocytic senescence may contribute to DA neuronal loss and PD progression.

Melatonin receptors MT1 (encoded by *MTNR1A*) and MT2 (*MTNR1B*) are G protein-coupled receptors (GPCRs) widely distributed in the brain (Lacoste et al., 2015). Melatonin, the endogenous ligand, exerts a wide range of biological functions through these receptors, including circadian rhythm modulation, anti-oxidation, and immunomodulation (Jia et al., 2025). Notably, our previous study indicated that MT1 expression is significantly reduced in the SNc of the PD mouse model, and genetic ablation of MT1 in primary microglia markedly exacerbated lipopolysaccharide (LPS)-induced inflammatory responses (Gu et al., 2021), suggesting a protective role of MT1 in neuroinflammation. Recent evidence also suggests that altered MT1/MT2 receptor signaling may contribute to neuroinflammatory and apoptotic processes in neurodegenerative models, highlighting a potential role for melatonin receptors in neuroprotection (Cimen et al., 2025). Consistently, melatonin has shown neuroprotective effects in PD models (Naskar et al., 2015; Su et al., 2015). However, whether melatonin receptor signaling regulates astrocytic senescence remains unexplored.

Ramelteon is a clinically approved MT1/MT2 agonist used for the treatment of insomnia (McGechan and Wellington, 2005; Kuriyama et al., 2014). It exhibits approximately 10-fold higher affinity for MT1 than for MT2 (Kato et al., 2005). Given its favorable safety profile and ability to cross the blood–brain barrier, Ramelteon warrants further investigation as a potential therapeutic agent in neurodegenerative diseases. Recent studies have demonstrated that Ramelteon exerts neuroprotective effects in various neurological disorders (Wang et al., 2018; Park et al., 2023; Zhang et al., 2023). Our previous work further showed that Ramelteon inhibited LPS-induced microglial activation and protected dopaminergic neurons against inflammatory damage *in vitro* and *in vivo* (Gu et al., 2021). However, whether Ramelteon regulates astrocytic senescence has not been investigated.

In this study, we investigated whether Ramelteon could inhibit astrocytic senescence and protect dopaminergic neurons, and whether this effect was specifically mediated by MT1. Our results demonstrated that Ramelteon suppressed both replicative and MPP^+^-induced astrocytic senescence through MT1-dependent cyclic adenosine monophosphate (cAMP) reduction and mitochondrial ROS (mitoROS) suppression, and this protective effect was recapitulated in an MPTP-induced mouse model of PD. Collectively, these findings establish Ramelteon as a potential therapeutic agent for PD by targeting MT1 to suppress astrocytic senescence and protect dopaminergic neurons.

## 2 Materials and methods

### 2.1 Animal experiments

A total of 84 C57BL/6 mice were used in this study, obtained from SLACCAL Lab Animal Ltd. (Shanghai, China). Of these, 44 male mice (6–8 weeks, 25–30 g) were used for the *in vivo* experiments, while 40 postnatal day 0–3 neonatal mice were used for primary astrocyte culture. These two cohorts were separate. For the *in vivo* experiments, mice were randomly assigned to four groups (*n* = 11 per group). Behavioral tests were performed with *n* = 8 mice per group; after behavioral assessment, 6 of these 8 mice were perfused and processed for TH immunohistochemistry. For immunoblot and qRT-PCR analyses, a separate cohort of *n* = 3 mice per group was used. Brain tissues (SNc) were collected, and each sample was divided into two portions for protein and RNA extraction, respectively. Male C57BL/6 mice were housed under a 12 h light/dark cycle with ad libitum access to food and water. All procedures were approved by the Animal Committee of Soochow University (Approval No. SUDA20250312A06).

For MPTP (1-methyl-4-phenyl-1,2,3,6-tetrahydropyridine)-induced PD model (Jackson-Lewis and Przedborski, 2007), mice were randomly assigned to four groups: vehicle + saline, Ramelteon + saline, vehicle + MPTP, and Ramelteon + MPTP. Ramelteon (20 mg/kg/day, i.p.; Selleck Chemicals, Houston, TX, United States) was administered for 14 consecutive days, beginning 7 days before MPTP injection and continuing during the 7-day MPTP treatment period. MPTP (30 mg/kg/day, i.p.; Sigma-Aldrich, St. Louis, MO, USA) was injected once daily for 7 days. Control groups received equal volumes of vehicle and/or saline. Vehicle consisted of 3% DMSO, 10% cremophor EL, and 87% D5W (Gu et al., 2021). Ramelteon was dissolved in vehicle and MPTP was dissolved in 0.9% saline. Mice were euthanized 24 h after the last injection. SNc were collected for protein, RNA extraction, or immunohistochemistry. A total of 44 male C57BL/6 mice were used in the *in vivo* experiments. Mice were randomly assigned to four groups (*n* = 11 per group). The allocation of animals was as follows: Behavioral tests (rotarod and pole test): *n* = 8 mice per group. After behavioral assessment, 6 of these 8 mice were perfused and processed for TH immunohistochemistry. Thus, the same animals were used for both behavioral tests and immunohistochemistry, but for different types of analysis. Immunoblot and qRT-PCR analyses: a separate cohort of *n* = 3 mice per group was used. Brain tissues (SNc) were collected, and each sample was divided into two portions: one for protein extraction (immunoblot) and one for RNA extraction (qRT-PCR).

### 2.2 Primary astrocyte culture and treatment

Primary astrocytes were isolated from postnatal day 0–3 C57BL/6 mouse cortices (SLACCAL Lab Animal Ltd., Shanghai, China) (McCarthy and de Vellis, 1980). Tissues were digested with 0.25% trypsin–EDTA for 15 min at 37 °C and seeded into poly-D-lysine-coated flasks. Cells were maintained in DMEM/F12 (Gibco) supplemented with 10% fetal bovine serum (FBS), 100 U/mL penicillin, and 100 μg/mL streptomycin. After 10–14 days, microglia were removed by shaking at 200 rpm for 2 h. A total of 40 neonatal mice were used. For each preparation, cortical tissues from 5 mice were pooled and seeded into one large culture dish. A total of 8 large dishes were prepared. These large dishes served as a source of astrocytes for subsequent experiments. For each experiment, astrocytes from one large dish were trypsinized, counted, and seeded into multiple wells of culture plates. Each well was independently treated (e.g., with Ramelteon, MPP^+^, siRNA, or vehicle).

For replicative senescence, astrocytes were cultured for 40 DIV (passaged every 4–5 days), with 10 DIV astrocytes serving as young controls. Senescent astrocytes were treated with Ramelteon (50 μM) for 5 days. For premature senescence, astrocytes (10 DIV) were pretreated with Ramelteon (50 μM) for 2 h, followed by MPP^+^ (500 μM) exposure for 24 h. The concentrations of Ramelteon (50 μM) and MPP^+^ (500 μM) were selected based on previous studies (Gu et al., 2021; Chen et al., 2024).

Astrocytes were pretreated with the adenylyl cyclase inhibitor SQ22536 (50 μM, HY-100396, MedChemExpress) for 30 min prior to Ramelteon or MPP^+^ stimulation. For cAMP rescue experiments, cells were co-treated with the cell-permeable cAMP analog dbcAMP (1 mM, HY-B0764, MedChemExpress) for 24 h. To scavenge mitochondrial ROS, cells were treated with the mitochondria-targeted antioxidant MitoQ (100 nM, HY-100116A, MedChemExpress) for 24 h.

### 2.3 Conditioned medium assay

MES23.5 cells, a dopaminergic (DA) cell line, were kindly gifted from Dr. Wei-Dong Le (Baylor College of Medicine, Houston, TX, United States), as previously described (Gu et al., 2017). Cells were cultured in DMEM/F12 supplemented with Sato’s components and 5% heat-inactivated fetal bovine serum, penicillin (100 U/mL), and streptomycin (100 μg/mL) at 37 °C in a humidified atmosphere containing 5% CO₂. For conditioned medium experiments, astrocytes were treated as indicated, and the media were collected after 24 h. MES23.5 cells were then cultured with the collected conditioned media for 24 h. Cell death was assessed by Hoechst/PI staining, and cytotoxicity was quantified by LDH release assay.

### 2.4 RNA interference

Astrocytes were transfected with siRNAs targeting mouse *Mtnr1a* (MEL-1A-R siRNA (m), sc-40113, Santa Cruz Biotechnology) or *Mtnr1b* (MEL-1B-R siRNA (m), sc-149365, Santa Cruz Biotechnology). Negative control siRNA (Santa Cruz Biotechnology) served as control. Transfections were performed using RNAiMAX (Invitrogen) according to the manufacturer‘s instructions. After 48 h, cells were treated with Ramelteon or vehicle. Knockdown efficiency was validated by quantitative RT-PCR using Santa Cruz primers (sc-40113-PR for *Mtnr1a*; sc-149365-PR for *Mtnr1b*).

### 2.5 SA-β-galactosidase staining

SA-β-gal staining was performed using a Senescence Cells Staining Kit (Beyotime, Shanghai, China) according to the manufacturer‘s instructions. Cells were fixed for 15 min at room temperature, incubated with staining solution at 37 °C overnight (pH 6.0), and photographed under a light microscope.

### 2.6 cAMP measurement

Intracellular cAMP levels were measured using a cAMP ELISA Kit (Abcam, Cambridge, UK) according to the manufacturer‘s instructions. Briefly, astrocytes were lysed in 0.1 M HCl, and the supernatants were collected for assay. cAMP levels were normalized to protein concentration.

### 2.7 Mitochondrial ROS measurement

Mitochondrial ROS were detected using MitoSOX Red (Invitrogen). Astrocytes were incubated with 2.5 μM MitoSOX for 15 min at 37 °C, washed twice with PBS, and fluorescence was measured using a fluorescence microscope (Olympus IX71) or flow cytometer (Cytek Aurora).

### 2.8 Immunoblot analysis

Cells or brain tissues were lysed in RIPA buffer containing protease and phosphatase inhibitors (Roche, Mannheim, Germany). Protein concentrations were determined using a BCA assay (Thermo Fisher Scientific). Equal amounts of protein (20 μg) were separated by SDS-PAGE and transferred to PVDF membranes (Millipore). Membranes were incubated with primary antibodies overnight at 4 °C, followed by HRP-conjugated secondary antibodies (Thermo Fisher Scientific). Proteins were visualized using ECL detection kits (Thermo Fisher Scientific). Primary antibodies used: anti-p16^Ink4a^ (1:1000, Proteintech, 10,883-1-AP), anti-LaminB1 (1:1000, Proteintech, 12,987-1-AP), anti-β-actin (1:5000, Abcam, ab8226).

### 2.9 RNA extraction and quantitative RT-PCR

Total RNA was extracted using TRIzol reagent (Invitrogen) and reverse-transcribed using PrimeScript RT Master Mix (Takara, Japan). Quantitative PCR was performed using SYBR Green Master Mix (Applied Biosystems) on an ABI 7500 Real-Time PCR System. Relative gene expression was calculated using the 2^−ΔΔCT^ method with β-actin as the internal control. The primer sequences were as follows: mouse *β-actin*: 5′-GACCTGACTGACTACCTC-3′ and 5′-GACAGCGAGGCCAGGATG-3′; *IL-6*: 5′-GCTATGAAGTTCCTCTCTGC-3′ and 5′-CTAGGTTTGCCGAGTAGATC-3′; *IL-1β*: 5′-TGGCAACTGTTCCTG-3′ and 5′-GGAAGCAGCCCTTCATCTTT-3′; *MMP-9*: 5′-CGACTTTTGTGGTCTTCCCC-3′ and 5′-AGCGGTACAAGTATGCCTCTG-3′; *CXCL10*: 5′-CCAAGTGCTGCCGTCATTTTC-3′ and 5′-GGCTCGCAGGGATGATTTCAA-3′. The primers for *Mtnr1a* (sc-40113-PR) and *Mtnr1b* (sc-149365-PR) were purchased from Santa Cruz Biotechnology.

### 2.10 Immunofluorescence and immunohistochemistry

For cell immunofluorescence, astrocytes were fixed with 4% paraformaldehyde (PFA), permeabilized with 0.25% Triton X-100, and blocked with 5% bovine serum albumin (BSA). Cells were incubated with primary antibodies overnight at 4 °C, followed by Alexa Fluor-conjugated secondary antibodies (Invitrogen). Nuclei were stained with DAPI (Sigma). For tissue immunohistochemistry, mice were perfused with 4% PFA, brains post-fixed and cryoprotected in 30% sucrose. Coronal sections (20 μm) of the midbrain were cut using a cryostat (Leica CM1950). Sections were blocked with 5% normal goat serum and incubated with primary antibodies, followed by Alexa Fluor-conjugated secondary antibodies. Primary antibodies used: anti-GFAP (1:500, Cell Signaling Technology, 3,670), anti-TH (1:300, Millipore, AB9072), anti-LaminB1 (1:500, Proteintech, 12,987-1-AP).

### 2.11 Behavioral tests

Rotarod test: Mice were trained on a rotarod apparatus (SANS Biological Technology, Nanjing, China) at a constant speed of 4 rpm for 2 min. For testing, speed was accelerated from 4 to 40 rpm over 5 min, and the latency to fall was recorded. Each mouse performed three trials, and the average was calculated. Pole test: A wooden pole (50 cm height, 1 cm diameter) was placed in the home cage. Mice were placed head-down on the top of the pole, and the time to descend to the cage floor was recorded. The test was repeated three times, and the average was calculated.

### 2.12 Statistical analysis

Statistical analyses were performed using one-way ANOVA followed by Tukey’s post-hoc test for single-factor comparisons with three or more groups. All analyses were performed using GraphPad Prism 8.0. A *p* value < 0.05 was considered statistically significant. Exact adjusted *p* values for all comparisons are provided in Supplementary Table S1.

## 3 Results

### 3.1 Ramelteon inhibits astrocytic senescence and alleviates DA neurodegeneration in MPTP-induced PD mouse model

To evaluate the therapeutic potential of Ramelteon *in vivo*, we employed the MPTP-induced mouse model of PD. Mice received daily intraperitoneal injections of Ramelteon (20 mg/kg) for 14 days before and during MPTP administration (Supplementary Figure S1A). Immunohistochemical staining revealed that MPTP treatment induced severe loss of TH^+^ dopaminergic neurons in the SNc, which was markedly attenuated by Ramelteon pretreatment (Figures 1A,B). Motor function was assessed using behavioral tests. In the rotarod test, MPTP-treated mice exhibited significantly reduced latency to fall, whereas Ramelteon pretreatment markedly improved motor performance (Figure 1C). Similarly, the pole test revealed that MPTP-induced increase in descending time was significantly reversed by Ramelteon treatment (Figure 1D). These results demonstrate that Ramelteon protects against MPTP-induced dopaminergic neuronal loss and motor deficits.

**Figure 1:**
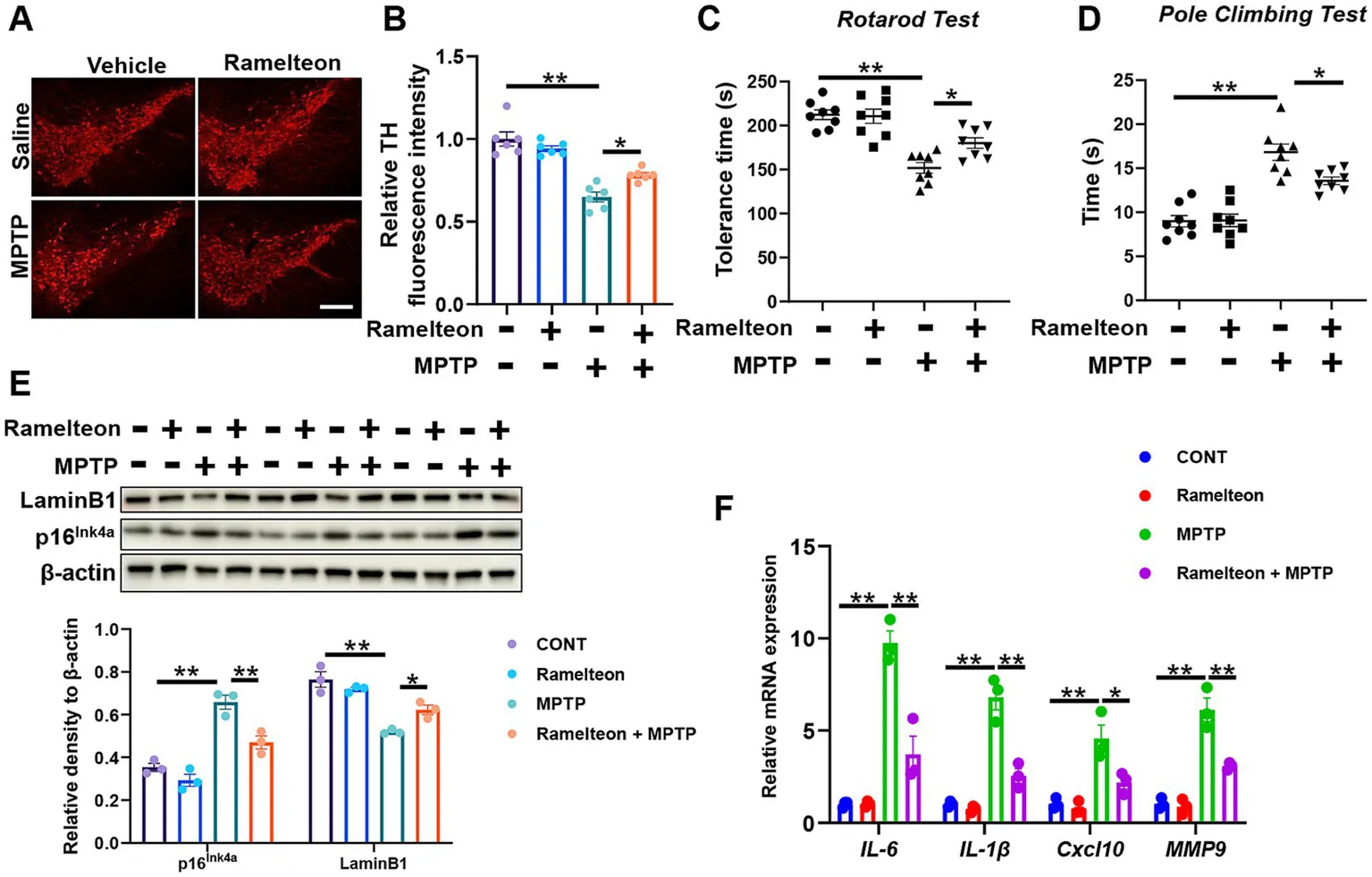
Ramelteon inhibits astrocytic senescence and alleviates DA neurodegeneration in MPTP-induced PD mouse model. **(A)** Representative images of TH immunohistochemical staining in the SNc of mice from the indicated groups. *n* = 6. Scale bar, 100 μm. **(B)** Quantification of TH^+^ neuron numbers in the SNc. **(C)** Rotarod test latency to fall. *n* = 8. **(D)** Pole test descending time. *n* = 8. **(E)** Immunoblot analysis of p16^Ink4a^, LaminB1, and β-actin in SNc tissues. *n* = 3. **(F)** qRT-PCR analysis of *IL-6*, *IL-1β*, *CXCL10*, and *MMP9* mRNA levels in the midbrain. *n* = 3. Data are presented as mean ± SEM. *p < 0*.05*, **p < 0.01; ns, not significant. One-way ANOVA followed by Tukey’s post-hoc test.

Given the emerging role of astrocytic senescence in PD pathogenesis (Xia et al., 2020; Chen et al., 2024), we next examined whether Ramelteon could suppress astrocytic senescence *in vivo*. Immunoblot analysis showed that MPTP challenge markedly upregulated the senescence marker p16^Ink4a^ and downregulated LaminB1 in the SNc, both of which were significantly reversed by Ramelteon treatment (Figure 1E). Furthermore, MPTP-induced elevation of SASP factors, including *IL-6*, *IL-1β*, *CXCL10*, and *MMP9*, were substantially suppressed by Ramelteon (Figure 1F). Collectively, these findings demonstrate that Ramelteon inhibits astrocytic senescence while concurrently protecting dopaminergic neurons in the MPTP-induced PD mouse model.

### 3.2 Ramelteon suppresses replicative astrocytic senescence *in vitro*

To determine whether Ramelteon directly regulates astrocytic senescence, a replicative senescence model was established by culturing primary astrocytes for 40 days *in vitro* (DIV) (Xia et al., 2020; Chen et al., 2024). Compared with astrocytes cultured for 10 DIV, 40 DIV astrocytes exhibited characteristic senescent phenotypes, as evidenced by increased SA-β-gal activity, enhanced p16^Ink4a^ protein expression, and reduced LaminB1 levels (Figures 2A–F).

**Figure 2:**
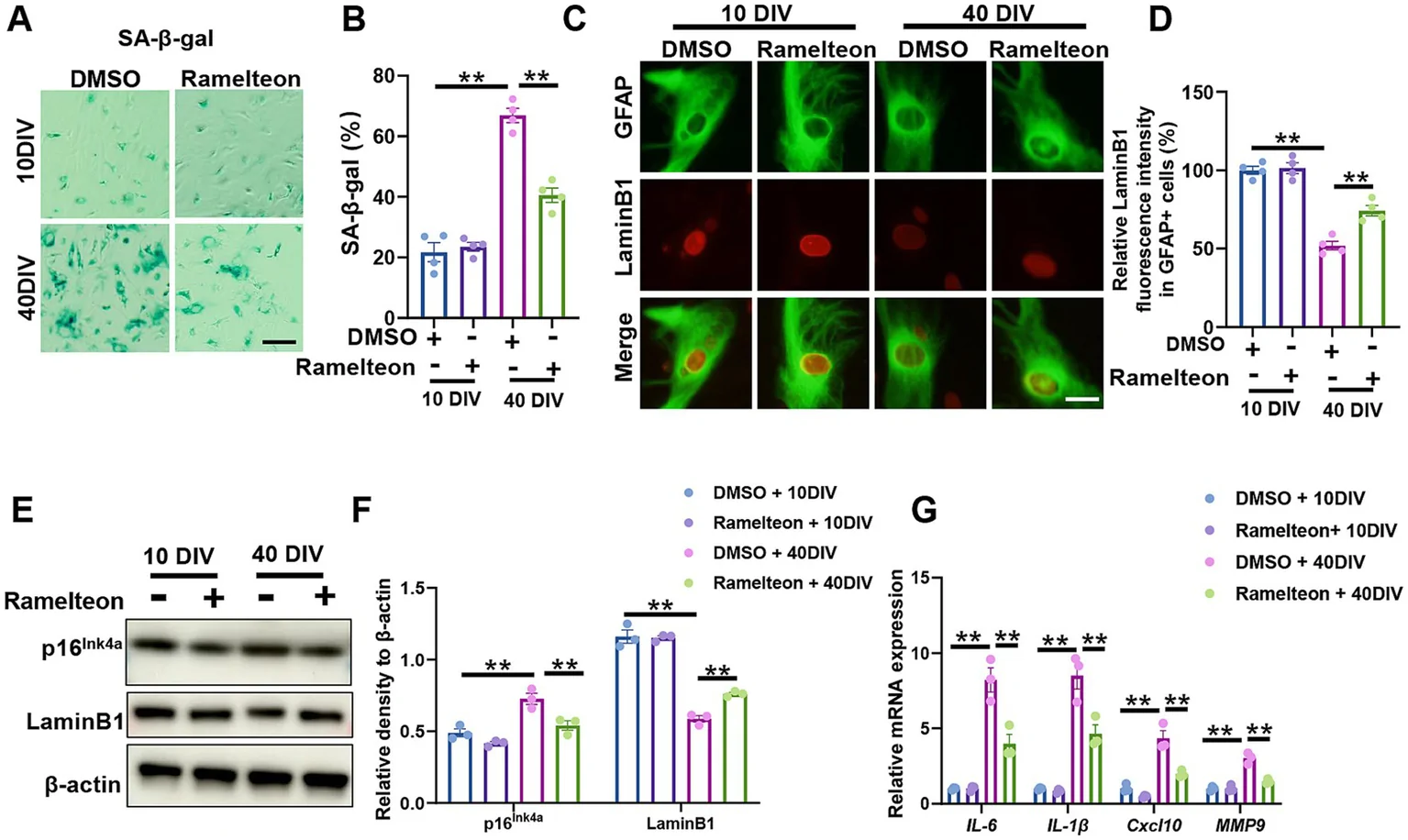
Ramelteon suppresses replicative astrocytic senescence *in vitro*. **(A)** Representative images of SA-β-gal staining in primary astrocytes cultured for 10 DIV or 40 DIV, treated with or without Ramelteon (50 μM) for 5 days. *n* = 4. Scale bar, 50 μm. **(B)** Quantification of SA-β-gal-positive cells. **(C)** Representative immunofluorescence images of GFAP (red) and LaminB1 (green) in astrocytes from the indicated groups. *n* = 4. Scale bar, 20 μm. **(D)** Quantification of LaminB1 fluorescence intensity in GFAP^+^ cells. **(E)** Immunoblot analysis of p16^Ink4a^, LaminB1, and β-actin. *n* = 3. **(F)** Quantification of protein levels normalized to β-actin. **(G)** qRT-PCR analysis of SASP factors (*IL-6*, *IL-1β*, *CXCL10*, and *MMP9*). *n* = 3. Data are presented as mean ± SEM. **p < 0*.01*. One-way ANOVA followed by Tukey’s post-hoc test.

Senescent astrocytes (40 DIV) were then treated with Ramelteon (50 μM) for 5 days. SA-β-gal staining demonstrated that Ramelteon significantly reduced the percentage of SA-β-gal-positive cells (Figures 2A,B). Immunofluorescence staining further revealed that Ramelteon treatment markedly increased LaminB1 fluorescence intensity in glial fibrillary acidic protein (GFAP)-positive astrocytes (Figures 2C,D). Consistently, immunoblot analysis confirmed that Ramelteon suppressed p16^Ink4a^ expression and restored LaminB1 protein levels in senescent astrocytes (Figures 2E,F). In addition to senescence marker expression, the SASP factors were assessed. Senescent astrocytes exhibited significantly elevated mRNA levels of pro-inflammatory SASP factors, including *IL-6*, *IL-1β*, *CXCL10*, and *MMP9* (Figure 2G). Notably, Ramelteon substantially attenuated the upregulation of these SASP factors (Figure 2G). Collectively, these results demonstrate that Ramelteon effectively suppresses replicative astrocytic senescence *in vitro*.

### 3.3 Ramelteon alleviates MPP^+^-induced premature astrocytic senescence

MPP^+^ (1-methyl-4-phenylpyridinium), the active metabolite of MPTP, is a well-established neurotoxin that induces mitochondrial dysfunction and cellular senescence in astrocytes (Wu et al., 1992; Xia et al., 2020). To determine whether Ramelteon could counteract neurotoxin-elicited premature senescence, primary astrocytes were pretreated with Ramelteon (50 μM) for 2 h prior to MPP^+^ (500 μM) exposure for 24 h. SA-β-gal staining showed that MPP^+^ challenge significantly increased the percentage of senescent cells, whereas Ramelteon pretreatment markedly reduced this effect (Figures 3A,B). Immunofluorescence staining further revealed that MPP^+^-induced reduction in LaminB1 in GFAP^+^ astrocytes was substantially restored by Ramelteon treatment (Figures 3C,D). Consistently, immunoblot analysis confirmed that Ramelteon reversed MPP^+^-induced upregulation of p16^Ink4a^ and downregulation of LaminB1 (Figures 3E,F). Moreover, MPP^+^-elicited elevation of SASP factors, including *IL-6*, *IL-1β*, *CXCL10*, and *MMP9*, was substantially attenuated by Ramelteon at the transcriptional level (Figure 3G). Collectively, these findings demonstrate that Ramelteon effectively mitigates MPP^+^-induced premature astrocytic senescence *in vitro*.

**Figure 3:**
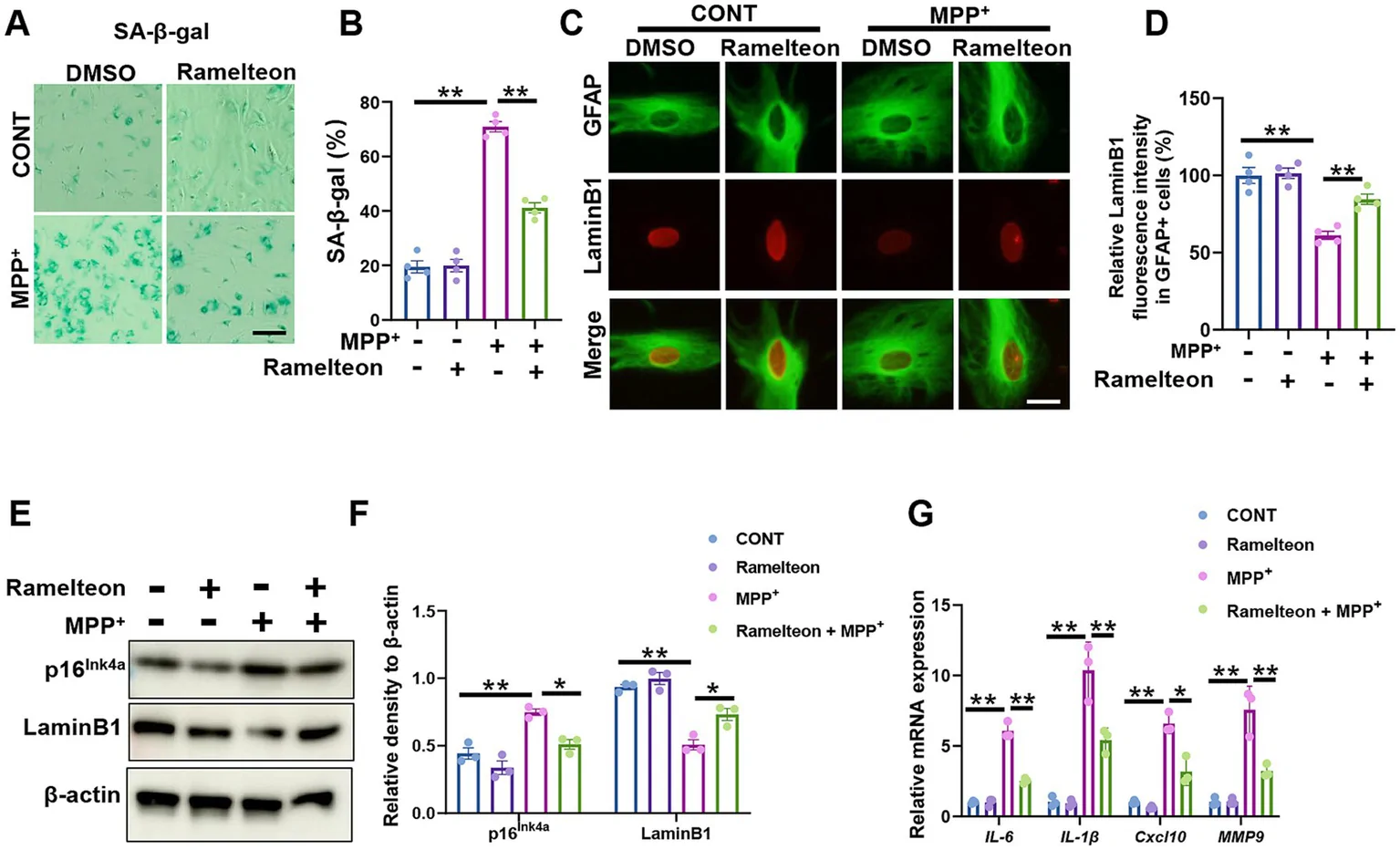
Ramelteon alleviates MPP^+^-induced premature astrocytic senescence. (**A)** Representative images of SA-β-gal staining in primary astrocytes pretreated with Ramelteon (50 μM) for 2 h followed by MPP^+^ (500 μM) exposure for 24 h. Scale bar, 50 μm. *n* = 4. **(B)** Quantification of SA-β-gal-positive cells. **(C)** Representative immunofluorescence images of GFAP (red) and LaminB1 (green). *n* = 4. Scale bar, 20 μm. **(D)** Quantification of LaminB1 fluorescence intensity in GFAP^+^ cells. **(E)** Immunoblot analysis of p16^Ink4a^, LaminB1, and β-actin. *n* = 3. **(F)** Quantification of protein levels normalized to β-actin. **(G)** qRT-PCR analysis of SASP factors (*IL-6*, *IL-1β*, *CXCL10*, and *MMP9*). *n* = 3. Data are presented as mean ± SEM. *p < 0*.05*, **p < *0.01*. One-way ANOVA followed by Tukey’s post-hoc test.

### 3.4 Ramelteon attenuates the neurotoxicity of senescent astrocytes

To assess whether Ramelteon could mitigate the neurotoxicity of senescent astrocytes, conditioned medium (CM) experiments were performed. Primary astrocytes subjected to either replicative senescence (40 DIV) or MPP^+^-induced premature senescence were treated with or without Ramelteon, and the resulting CM was collected to culture MES23.5 dopaminergic neuronal cells (Figure 4A).

**Figure 4:**
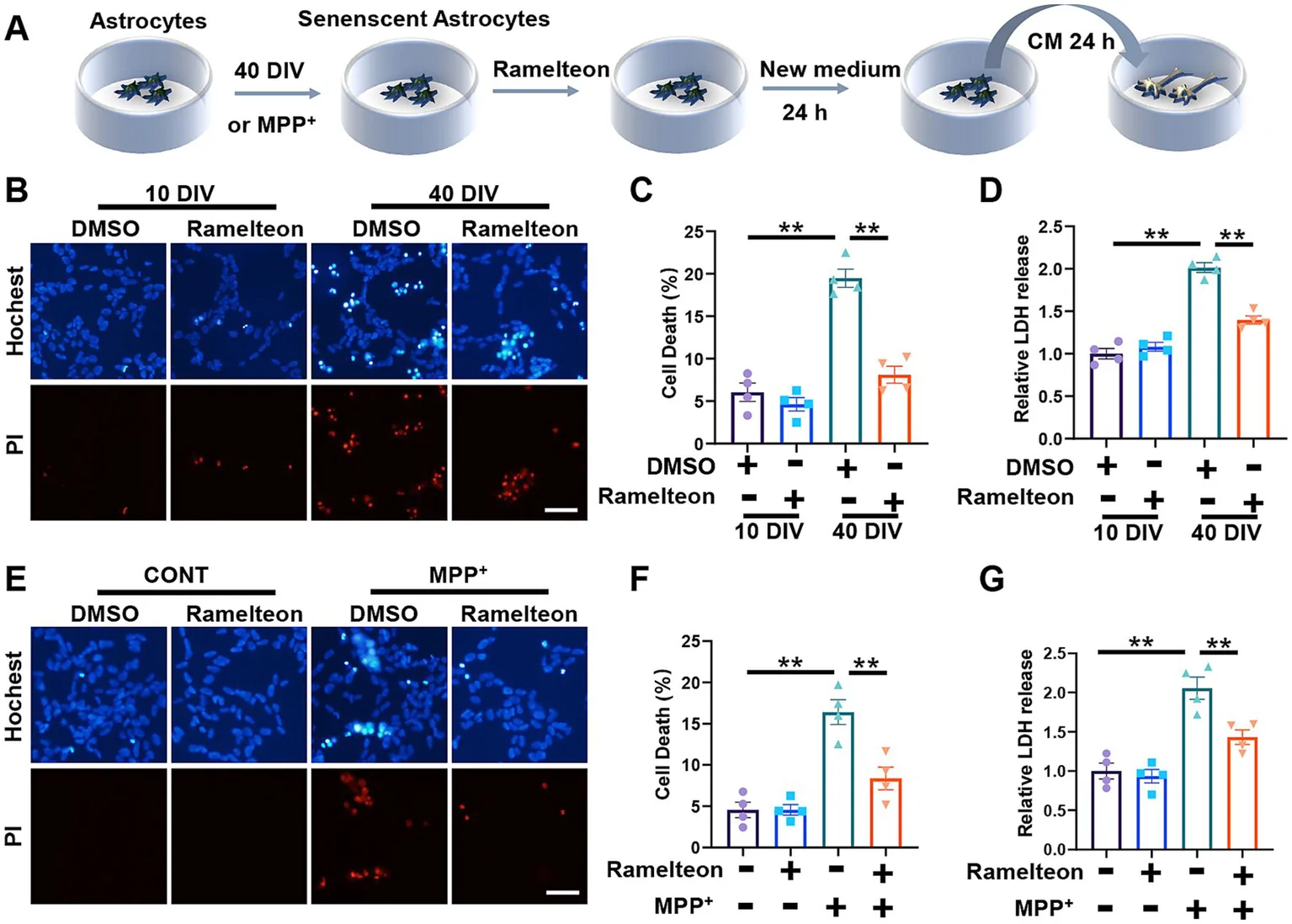
Ramelteon attenuates the neurotoxicity of senescent astrocytes. **(A)** Schematic illustration of the conditioned medium (CM) transfer experiment. Astrocytes subjected to replicative senescence (40 DIV) or MPP^+^-induced senescence were treated with or without Ramelteon, and the CM was collected to culture MES23.5 cells for 24 h. **(B)** Representative images of Hoechst (blue) and PI (red) staining of MES23.5 cells cultured with CM from 40 DIV astrocytes. *n* = 4. Scale bar, 50 μm. **(C)** Quantification of PI-positive cells. **(D)** LDH release assay of MES23.5 cells cultured with CM from 40 DIV astrocytes. *n* = 4. **(E)** Representative images of Hoechst/PI staining of MES23.5 cells cultured with CM from MPP^+^-treated astrocytes. Scale bar, 50 μm. *n* = 4. **(F)** Quantification of PI-positive cells. **(G)** LDH release assay of MES23.5 cells cultured with CM from MPP^+^-treated astrocytes. *n* = 4. Data are presented as mean ± SEM. **p < 0*.01*. One-way ANOVA followed by Tukey’s post-hoc test.

In the replicative senescence model, CM from 40 DIV senescent astrocytes markedly increased the percentage of propidium iodide (PI)-positive cells and lactate dehydrogenase (LDH) release in MES23.5 cells, indicating enhanced cytotoxicity (Figures 4B–D). In contrast, CM derived from Ramelteon-treated senescent astrocytes significantly reduced both PI positivity and LDH release (Figures 4B–D). Similarly, in the MPP^+^-induced senescence model, CM from MPP^+^-treated astrocytes conferred obvious neurotoxicity, whereas Ramelteon pretreatment substantially attenuated this detrimental effect, as evidenced by decreased PI-positive cells and reduced LDH levels (Figures 4E–G). Collectively, our results demonstrate that Ramelteon treatment effectively suppresses the neurotoxic capacity of senescent astrocytes toward dopaminergic neurons.

### 3.5 MT1 is required for the anti-senescence effects of Ramelteon in astrocytes

Given that Ramelteon is a non-selective agonist for both MT1 and MT2 receptors, we next sought to determine which receptor mediates its anti-senescence effects. Primary astrocytes were transfected with specific siRNAs targeting *Mtnr1a* (encoding MT1) or *Mtnr1b* (encoding MT2), with knockdown efficiency confirmed by quantitative real-time PCR (qPCR) (Supplementary Figure S1B). In the replicative senescence model (40 DIV), *Mtnr1a* knockdown substantially abolished Ramelteon-induced reduction of p16^Ink4a^ and restoration of LaminB1 (Figures 5A,B). In contrast, *Mtnr1b* knockdown did not appreciably affect Ramelteon-mediated regulation of p16^Ink4a^ or LaminB1 expression (Figures 5E,F). Similarly, in the MPP^+^-induced senescence model, *Mtnr1a* knockdown largely abolished the protective effects of Ramelteon, as evidenced by the failure to reverse MPP^+^-induced upregulation of p16^Ink4a^ and downregulation of LaminB1 (Figures 5C,D). By contrast, *Mtnr1b* knockdown did not influence Ramelteon’s effects on these senescence-associated markers (Figures 5G,H), with no significant alterations in p16^Ink4a^ and LaminB1 levels. Collectively, these results showed that the anti-senescence effects of Ramelteon are specifically mediated by MT1.

**Figure 5:**
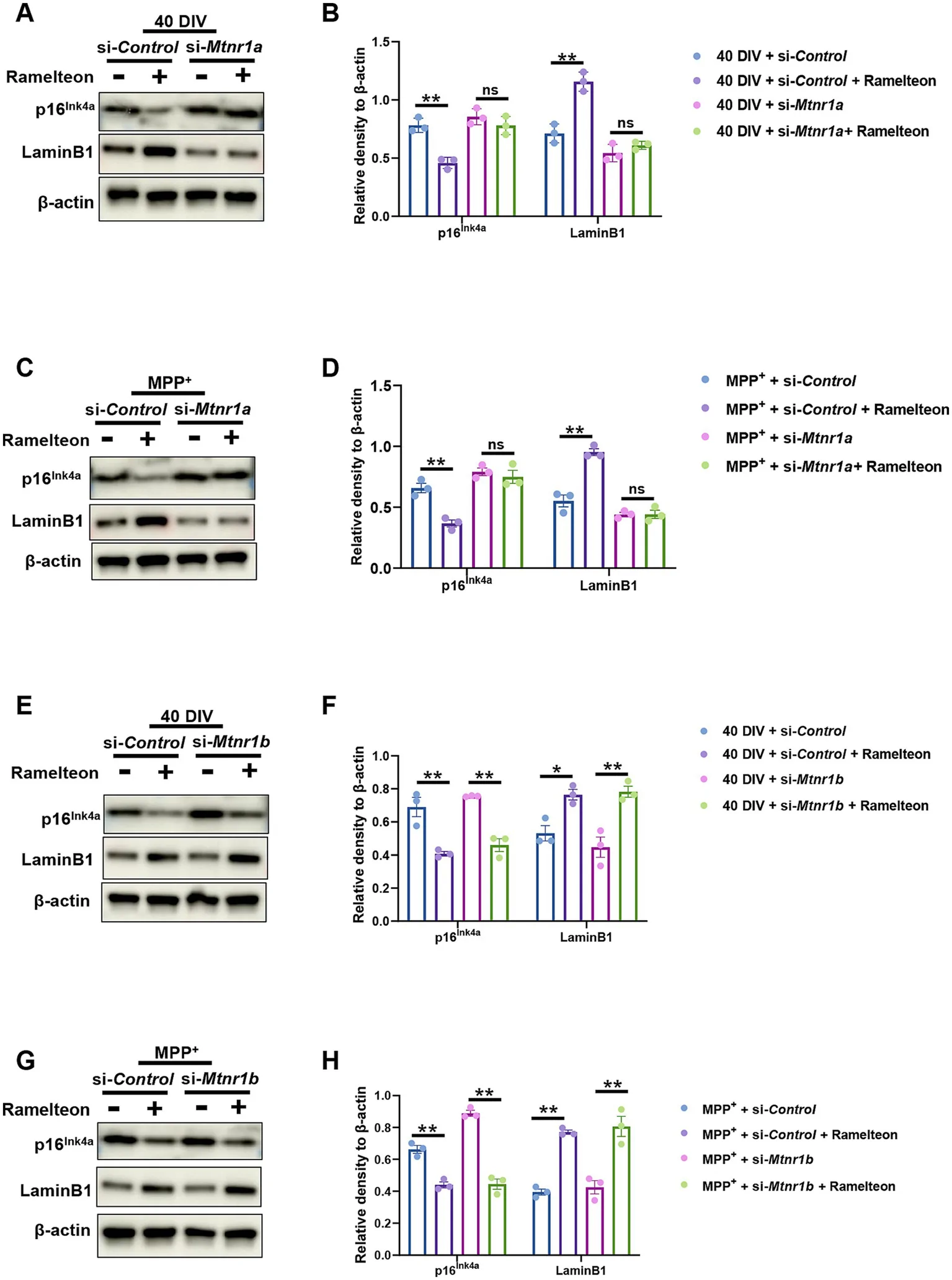
MT1 is required for the anti-senescence effects of Ramelteon in astrocytes. **(A)** Immunoblot analysis of p16^Ink4a^, LaminB1, and β-actin in 40 DIV astrocytes transfected with si-*Control* or si-*Mtnr1a* and treated with Ramelteon (50 μM) for 5 days. **(B)** Quantification of protein levels normalized to β-actin. **(C)** Immunoblot analysis of p16^Ink4a^, LaminB1, and β-actin in MPP^+^-treated astrocytes transfected with si-*Control* or si-*Mtnr1a*. *n* = 3. **(D)** Quantification of protein levels normalized to *β*-actin. **(E)** Immunoblot analysis of p16^Ink4a^, LaminB1, and *β*-actin in 40 DIV astrocytes transfected with si-*Control* or si-*Mtnr1b*. **(F)** Quantification of protein levels normalized to *β*-actin. **(G)** Immunoblot analysis of p^16Ink4a^, LaminB1, and *β*-actin in MPP^+^-treated astrocytes transfected with si-*Control* or si-*Mtnr1b*. **(H)** Quantification of protein levels normalized to *β*-actin. Data are presented as mean ± SEM. *n* = 3. *p < 0*.05*, **p < *0.01*; ns, not significant. One-way ANOVA followed by Tukey’s post-hoc test.

### 3.6 cAMP reduction contributes to Ramelteon-induced suppression of astrocytic senescence

MT1 is a classical Gαi/o-coupled receptor, and its activation leads to the inhibition of adenylate cyclase (AC) and subsequent reduction of intracellular cAMP levels (Liu et al., 2015). To investigate whether cAMP signaling mediates the anti-senescence effect of Ramelteon, cAMP levels were measured under various conditions. Senescent astrocytes (40 DIV) exhibited significantly higher cAMP levels compared to young astrocytes (10 DIV). Ramelteon treatment not only suppressed basal cAMP production in 10 DIV astrocytes, but also markedly attenuated the aberrant cAMP elevation induced by replicative senescence in 40 DIV astrocytes (Figure 6A). Consistently, in the MPP^+^-induced senescence model, MPP^+^ challenge led to an increase in cAMP levels, which was substantially reversed by Ramelteon co-treatment (Figure 6B).

**Figure 6:**
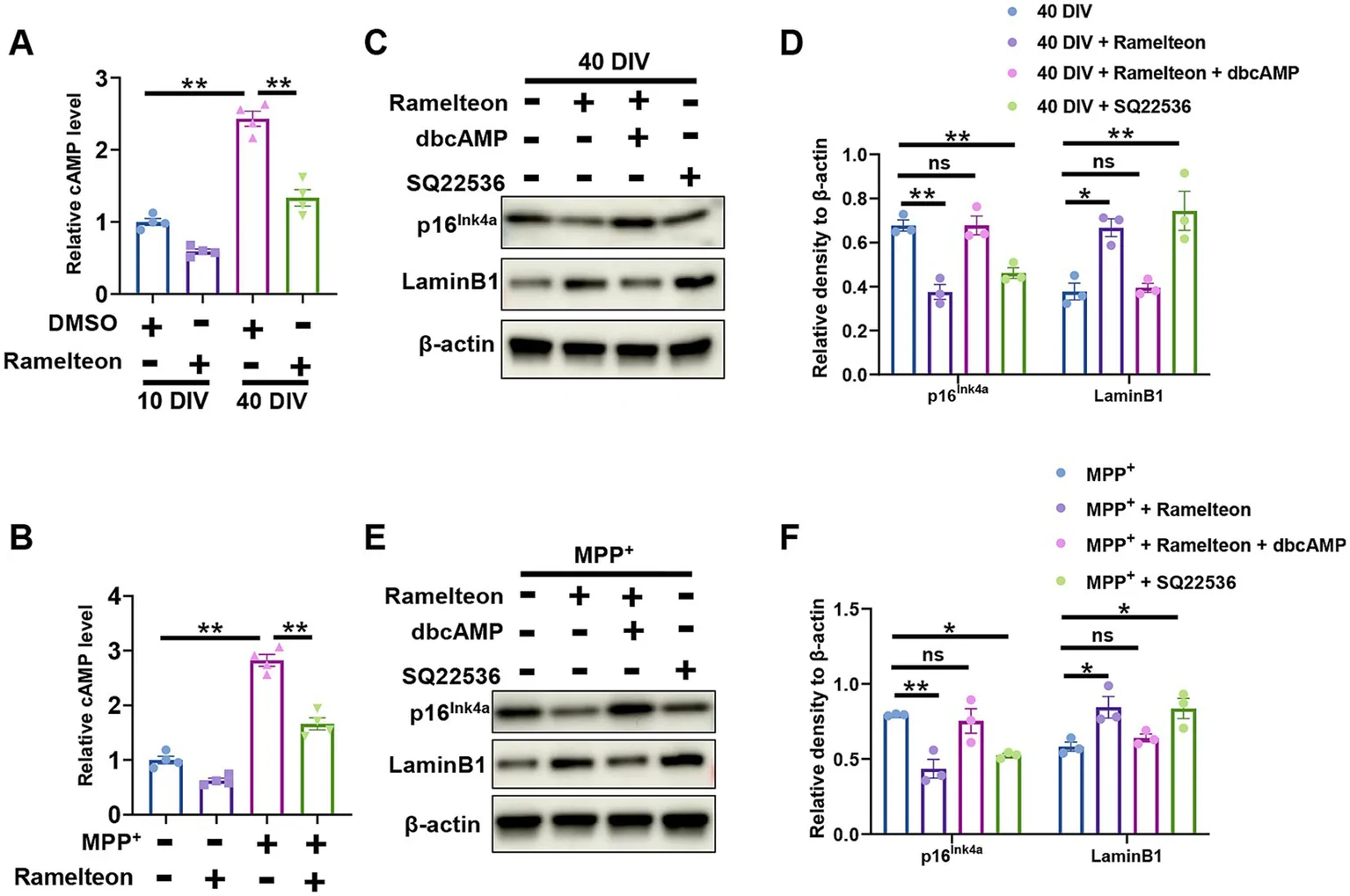
cAMP reduction contributes to Ramelteon-induced suppression of astrocytic senescence. **(A)** Intracellular cAMP levels in 10 DIV and 40 DIV astrocytes treated with or without Ramelteon (50 μM) for 5 days. *n* = 4. **(B)** Intracellular cAMP levels in astrocytes treated with MPP^+^ (500 μM) in the presence or absence of Ramelteon (50 μM). *n* = 4. **(C)** Immunoblot analysis of p16^Ink4a^, LaminB1, and *β*-actin in 40 DIV astrocytes treated with Ramelteon and/or SQ22536 (50 μM) or dbcAMP (1 mM). *n* = 3. **(D)** Quantification of protein levels normalized to β-actin. **(E)** Immunoblot analysis of p16^Ink4a^, LaminB1, and *β*-actin in MPP^+^-treated astrocytes with Ramelteon and/or SQ22536 (50 μM) or dbcAMP (1 mM). *n* = 3. **(F)** Quantification of protein levels normalized to β-actin. *p < 0*.05*, **p < 0*.01*. One-way ANOVA followed by Tukey’s post-hoc test.

To determine whether the reduction of cAMP contributes to the anti-senescence effect of Ramelteon, SQ22536, a specific AC inhibitor, was employed. Treatment with SQ22536 alone significantly decreased p16^Ink4a^ expression and increased LaminB1 levels in senescent astrocytes (40 DIV) (Figures 6C,D). In the MPP^+^ model, SQ22536 similarly reversed MPP^+^-induced upregulation of p16^Ink4a^ and downregulation of LaminB1 (Figures 6E,F), further supporting that cAMP reduction is sufficient to suppress astrocytic senescence. Conversely, dbcAMP, a cell-permeable cAMP analog, was used to evaluate whether restoring cAMP levels could counteract the protective effect of Ramelteon. In both 40 DIV and MPP^+^ models, dbcAMP treatment substantially reversed Ramelteon-induced downregulation of p16^Ink4a^ and upregulation of LaminB1 (Figures 6C–F), indicating that sustained cAMP elevation abolishes the anti-senescence effect of Ramelteon. Collectively, these findings demonstrate that Ramelteon suppresses astrocytic senescence through attenuation of aberrant cAMP elevation.

### 3.7 MT1-cAMP signaling regulates mitochondrial ROS production in senescent astrocytes

Excessive production of mitoROS has been recognized as a critical driver of cellular senescence (Ziegler et al., 2014). Emerging evidence indicates that aberrant cAMP elevation promotes mitochondrial oxidative stress (Richartz et al., 2022; Qian et al., 2023). To determine whether MT1-cAMP signaling modulates mitoROS production, MitoSOX staining was performed. Senescent astrocytes (40 DIV) exhibited markedly elevated mitoROS levels compared to young controls. Treatment with Ramelteon significantly attenuated this increase, and a similar effect was observed with SQ22536 (Figure 7A). Quantitative analysis by flow cytometry further confirmed these findings (Figures 7B,C). Consistently, in the MPP^+^-induced premature senescence model, both Ramelteon and SQ22536 effectively suppressed MPP^+^-elicited mitoROS overproduction (Figures 7D–F).

**Figure 7:**
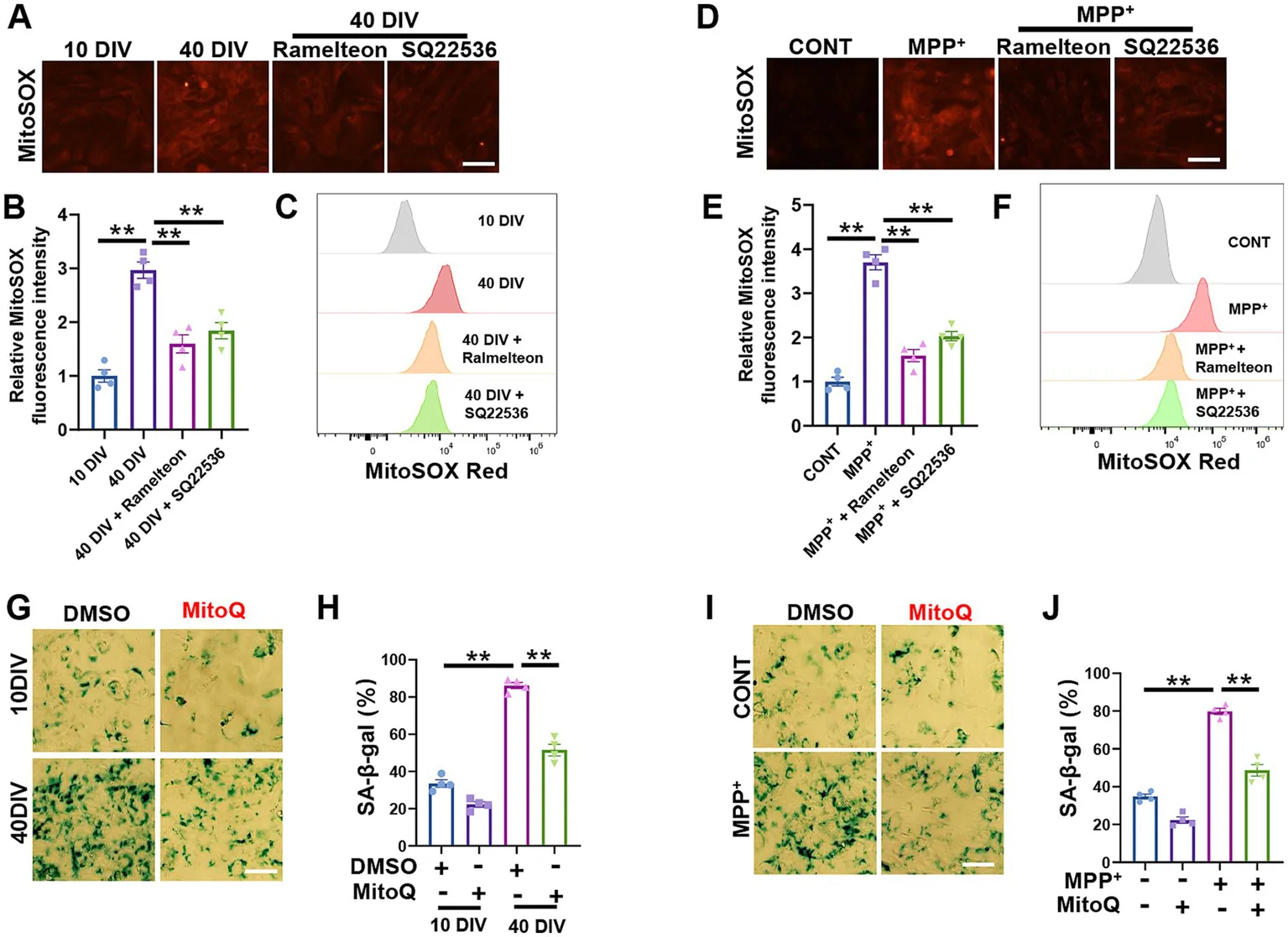
MT1-cAMP signaling regulates mitochondrial ROS production in senescent astrocytes. (A) Representative images of MitoSOX staining in 40 DIV astrocytes treated with Ramelteon (50 μM) or SQ22536 (50 μM) for 5 days. Scale bar, 20 μm. *n* = 4. **(B)** Quantification of MitoSOX fluorescence intensity of **(A)**. (C) Flow cytometry analysis of MitoSOX fluorescence intensity. **(D)** Representative images of MitoSOX staining in MPP^+^-treated astrocytes with Ramelteon or SQ22536. Scale bar, 20 μm. *n* = 4. **(E)** Quantification of MitoSOX fluorescence intensity of **(D)**. **(F)** Flow cytometry analysis of MitoSOX fluorescence intensity. **(G)** Representative images of SA-β-gal staining in 40 DIV astrocytes treated with mitoQ (100 nM) for 5 days. Scale bar, 50 μm. *n* = 4. **(H)** Quantification of SA-β-gal-positive cells. **(I)** Representative images of SA-β-gal staining in MPP^+^-treated astrocytes with mitoQ. Scale bar, 50 μm. *n* = 4. **(J)** Quantification of SA-β-gal-positive cells. Data are presented as mean ± SEM. **p < 0*.01*. One-way followed by Tukey’s post-hoc test.

To further determine whether mitoROS reduction contributes to the anti-senescence effect of Ramelteon, mitoQ, a mitochondria-targeted antioxidant, was employed. Treatment with mitoQ significantly reduced SA-β-gal positivity in both replicative (40 DIV) and MPP^+^-induced senescent astrocytes (Figures 7G–J), mimicking the effect of Ramelteon. Collectively, these data indicate that MT1-cAMP signaling suppresses astrocytic senescence through reduction of mitochondrial ROS production.

## 4 Discussion

In the present study, we provide evidence that Ramelteon suppresses astrocytic senescence to protect against dopaminergic neuronal loss in the MPTP-induced mouse model of PD. Mechanistically, we find that the MT1-cAMP-mitoROS axis may contribute to this effect (Figure 8). These findings suggest a previously unrecognized function of MT1 in regulating astrocytic senescence and provide a mechanistic basis for the neuroprotective effects of Ramelteon in PD. However, the conclusions should be interpreted within the context of the experimental models and the limitations discussed below.

**Figure 8:**
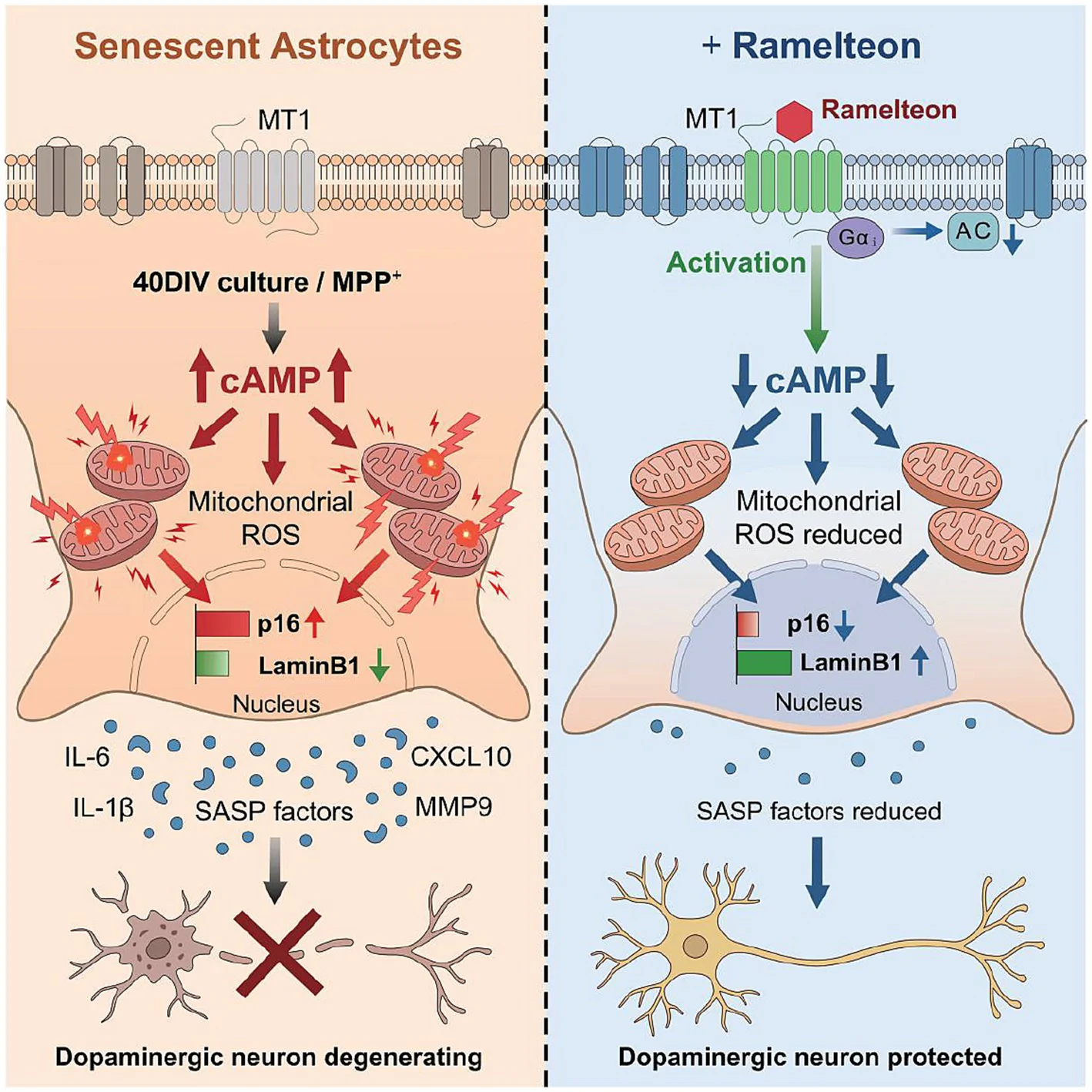
Schematic diagram illustrating the proposed mechanism by which Ramelteon suppresses astrocytic senescence and protects dopaminergic neurons. Ramelteon activates MT1 receptor coupled to Gα_i_ protein, which inhibits adenylate cyclase (AC) and reduces intracellular cAMP levels. The suppression of cAMP leads to decreased mitochondrial ROS production. This signaling cascade attenuates astrocytic senescence, as evidenced by reduced p16^Ink4a^ expression and SASP factor release, and ultimately protects dopaminergic neurons from degeneration. Created with BioRender.com.

Multiple evidences support a protective role of MT1 in PD (Gu et al., 2021; Lv et al., 2024; Yao et al., 2024). MT1 and MT2 expression is reduced in the substantia nigra of PD patients (Adi et al., 2010). Our previous work demonstrated that MT1 activation suppresses aerobic glycolysis and promotes oxidative phosphorylation in microglia (Gu et al., 2021). MT1 also modulates mitochondrial dynamics and promotes autophagy to mitigate α-synuclein aggregation in neuron (Yao et al., 2024; Wang et al., 2025). In astrocytes, our present data suggest that MT1 activation reduces mitochondrial ROS production. This discrepancy may reflect differences in cellular metabolism and inflammatory contexts. Recent studies have further elucidated the neuroprotective mechanisms of MT1. MT1 activation prevents α-synuclein-induced ferroptosis through the Sirt1/Nrf2/Ho-1/Gpx4 pathway (Lv et al., 2024). In a zebrafish PD model, melatonin improved motor and sleep–wake behavior in an MT1-dependent manner, and this effect was associated with restoration of lipid metabolism (Pang et al., 2024). These findings suggest that MT1 exerts neuroprotection through both direct neuronal effects and indirect modulation of glial cells, although cell-type-specific contributions remain to be fully defined.

A central finding of our study is the role of aberrant cAMP elevation in driving astrocytic senescence. Senescent astrocytes exhibited markedly elevated cAMP levels. Ramelteon reversed this elevation through MT1-dependent signaling. We further investigated the downstream effector of this pathway. Senescent astrocytes displayed elevated mitochondrial ROS production. The mitochondria-targeted antioxidant mitoQ recapitulated the anti-senescence effect of Ramelteon, confirming that mitochondrial ROS reduction contributes to senescence suppression. The relationship between cAMP and mitochondrial ROS is complex and context-dependent (Bouchez and Devin, 2019; Palmeira et al., 2019). Some studies have shown that cAMP promotes ROS production (Richartz et al., 2022; Qian et al., 2023), while others demonstrate that cAMP reduces ROS via protein kinase A (PKA)-dependent modulation of mitochondrial complex I (Signorile et al., 2022). These opposing effects likely reflect differences in cell types, pathological contexts, or engagement of distinct downstream pathways. In our astrocyte senescence models, cAMP elevation correlated with increased mitoROS production, and cAMP reduction suppressed this increase. Collectively, these data suggest a cohesive signaling cascade linking MT1 activation to mitochondrial redox homeostasis and senescence regulation.

Sleep disturbance is one of the most common nonmotor symptoms in PD, affecting up to 80% of patients (Zuzuárregui and During, 2020). Sleep fragmentation and circadian disruption may exacerbate PD pathology (Koc et al., 2025; Zafar and Schneider, 2026). Ramelteon is already approved for insomnia treatment and may therefore offer a dual benefit: correcting sleep–wake cycles while counteracting astrocytic senescence. Our results suggest that the anti-senescence effect is primarily mediated by MT1. *Mtnr1a* knockdown abolished Ramelteon’s protective effects, whereas *Mtnr1b* knockdown had no effect. This specificity is clinically relevant, as Ramelteon has approximately 10-fold higher affinity for MT1 than for MT2 (Kato et al., 2005). The recent elucidation of the MT1 receptor crystal structure (Stauch et al., 2019) may facilitate the rational design of MT1-selective agonists with enhanced therapeutic benefit and reduced off-target effects. Nevertheless, the clinical efficacy of Ramelteon in PD patients remains to be tested in prospective trials.

Several limitations define the scope of this study. The molecular analyses relied on a small sample size and only male mice, which may limit generalizability. The *in vivo* findings are based on the MPTP model, which reproduces acute dopaminergic loss but not the progressive and multifactorial nature of PD. Although siRNA knockdown supports MT1 involvement, astrocyte-specific conditional knockout mice were not used, leaving the cell-type-specific contribution of MT1 *in vivo* to be defined. Ramelteon is not entirely MT1-selective, and the vehicle contained Cremophor EL, although all groups received equal vehicle volumes. Finally, the link between cAMP reduction and mitochondrial ROS suppression was not fully delineated, and the 40 DIV model may not fully recapitulate the aging microenvironment. These limitations inform the interpretation of our findings and highlight directions for future studies.

## 5 Conclusion

In conclusion, our study establishes that Ramelteon-mediated MT1 activation suppresses astrocytic senescence through cAMP reduction and mitochondrial ROS suppression. These findings provide a mechanistic basis for the neuroprotective effects of Ramelteon in PD and support MT1 as a therapeutic target for age-related neurodegenerative diseases.

## Data Availability

The original contributions presented in the study are included in the article/Supplementary material, further inquiries can be directed to the corresponding authors.
