# Supplementary material for: Ramelteon suppresses astrocytic senescence via MT1-cAMP axis to protect dopaminergic neurons
Source: Front Aging Neurosci. 2026 Oct 26;18:1974509. doi: 10.3389/fnagi.2026.1974509 (PMC13745544; doi:10.3389/fnagi.2026.1974509)
Supplement: Supplementary file 1 [file Supplementary_file_1.docx]

**Supplementary Material**

**Figure S1**

**A**

**B**

**Figure S1.** (A) Schematic diagram of the animal experimental protocol. (B) Knockdown efficiency of si-*Mtnr1a* and si-*Mtnr1b* in primary astrocytes assessed by qRT-PCR. Data are presented as mean ± SEM. n = 3 independent experiments. ***p < 0.01* vs. si-*Control*. Student's t-test.
